# Chinese Herbal Medicine Treatment Improves the Overall Survival Rate of Individuals with Hypertension among Type 2 Diabetes Patients and Modulates *In Vitro* Smooth Muscle Cell Contractility

**DOI:** 10.1371/journal.pone.0145109

**Published:** 2015-12-23

**Authors:** Ying-Ju Lin, Tsung-Jung Ho, Yi-Chun Yeh, Chi-Fung Cheng, Yi-Tzone Shiao, Chang-Bi Wang, Wen-Kuei Chien, Jin-Hua Chen, Xiang Liu, Hsinyi Tsang, Ting-Hsu Lin, Chiu-Chu Liao, Shao-Mei Huang, Ju-Pi Li, Cheng-Wen Lin, Hao-Yu Pang, Jaung-Geng Lin, Yu-Ching Lan, Yu-Huei Liu, Shih-Yin Chen, Fuu-Jen Tsai, Wen-Miin Liang

**Affiliations:** 1 School of Chinese Medicine, China Medical University, Taichung, Taiwan; 2 Genetic Center, Department of Medical Research, China Medical University Hospital, Taichung, Taiwan; 3 Division of Chinese Medicine, China Medical University Beigang Hospital, Yunlin, Taiwan; 4 Division of Chinese Medicine, Tainan Municipal An-Nan Hospital-China Medical University, Tainan, Taiwan; 5 Graduate Institute of Biostatistics, School of Public Health, China Medical University, Taichung, Taiwan; 6 Heart Center, China Medical University Hospital, Taichung, Taiwan; 7 Biostatistics Center, College of Management, Taipei Medical University, Taipei, Taiwan; 8 School of Health Care Administration, College of Management, Taipei Medical University, Taipei, Taiwan; 9 National Institute of Allergy and Infectious Diseases, National Institutes of Health, Bethesda, Maryland, United States of America; 10 Rheumatism Research Center, China Medical University Hospital, Taichung, Taiwan; 11 Department of Medical Laboratory Science and Biotechnology, China Medical University, Taichung, Taiwan; 12 Department of Health Risk Management, China Medical University, Taichung, Taiwan; 13 Graduate Institute of Integrated Medicine, China Medical University, Taichung, Taiwan; 14 Asia University, Taichung, Taiwan; Cleveland Clinic Lerner Research Institute, UNITED STATES

## Abstract

Type 2 diabetes (T2D) is a chronic, multifactorial, and metabolic disorder accounting for 90% diabetes cases worldwide. Among them, almost half of T2D have hypertension, which is responsible for cardiovascular disease, morbidity, and mortality in these patients. The Chinese herbal medicine (CHM) prescription patterns of hypertension individuals among T2D patients have yet to be characterized. This study, therefore, aimed to determine their prescription patterns and evaluate the CHM effect. A cohort of one million randomly sampled cases from the National Health Insurance Research Database (NHIRD) was used to investigate the overall survival rate of CHM users, and prescription patterns. After matching CHM and non-CHM users for age, gender and date of diagnosis of hypertension, 980 subjects for each group were selected. The CHM users were characterized with slightly longer duration time from diabetes to hypertension, and more cases for hyperlipidaemia. The cumulative survival probabilities were higher in CHM users than in non-CHM users. Among these top 12 herbs, Liu-Wei-Di-Huang-Wan, Jia-Wei-Xiao-Yao-San, Dan-Shen, and Ge-Gen were the most common herbs and inhibited *in vitro* smooth muscle cell contractility. Our study also provides a CHM comprehensive list that may be useful in future investigation of the safety and efficacy for individuals with hypertension among type 2 diabetes patients.

## Introduction

Type 2 diabetes (T2D) is a chronic, multifactorial, and metabolic disorder and accounts for 90% of those with diabetes worldwide [1]. In Asia and the eastern Pacific region, China was home to the largest number of adults with diabetes (i.e. 90.0 million, or 9% of the population), followed by India (61.3 million, or 8% of the population) and Bangladesh (8.4 million, or 10% of the population) [2–4]. In Taiwan, T2D is one of the top 10 leading causes of death, suggesting that this disease is one of the most important health problems today. T2D is characterized by abnormally high levels of blood glucose resulting from impaired pancreatic β cell function, decreased insulin sensitivity in target tissues, and increased glucose output from the liver [5, 6]. Chronic hyperglycemia causes multiple organ damage and failure, affecting sites including the blood vessels and heart, eyes, kidneys, and nerves. Diabetes related cardiovascular disease, retinopathy, nephropathy, neuropathy, and peripheral circulatory disorders are believed to be responsible for the symptoms, signs, ill-defined secondary conditions, and mortality observed in patients with diabetes.

Hypertension (high blood pressure, usually > 130/80 mmHg) is very common among T2D patients. Almost half of T2D patients have high blood pressure, which doubles their risk of cardiovascular disease [7, 8]. Diabetes related cardiovascular diseases are believed to be responsible for the high morbidity and mortality rates of this condition [9]. In prospective studies, blood pressure control was twice as effective as glucose control in preventing diabetes related cardiovascular disease [10–13]. Therefore, both control of blood pressure and glucose levels in order to prevent substantial diabetic related complications and mortality continues to be an important public health concern.

In diabetic patients, significant improvements can be achieved by lifestyle modification [14] and treatment with hypoglycemic or anti-hyperglycemic, insulin sensitizing, and insulin secretion enhancing agents [15–17]. However, side effects are still frequently reported when using these therapeutic regimes. Meta-analyses show increased cardiovascular and mortality risk when using metformin, sulfonylurea, and thiazolidinediones [15–18]. Long-term thiazolidinedione use increases the risk of fracture, lower respiratory tract infection, and bladder cancer among those with diabetes [17, 19, 20]. These reports have prompted the search for alternative and complementary therapies for better management of diabetes and its related complications. Chinese herbal medicine (CHM) has been used in clinical practice for clinical, chronic, and irreversible diseases for hundreds of years. It has also been used in the management of diabetes, as well as diabetes related complications and mortality [21–23].

CHM is an important aspect of health care in Taiwan and is provided by licensed traditional Chinese medicine (TCM) doctors. It has also been covered under the National Health Insurance (NHI) program since 1996 [24, 25]. Residents in Taiwan are able to choose regular medical treatments, CHM, or both. All claims are collected by the National Health Insurance Research Database (NHIRD). Therefore, this claim database can be used as a platform to explore the utilization and therapeutic effects of Chinese herbal therapies prescribed by these TCM doctors in Taiwan. The characteristics of TCM use in Taiwan have been investigated by population-based studies for several diseases including childhood asthma [26], breast cancer [27], chronic kidney disease [28], diabetes [29], endometriosis [30], primary dysmenorrhea [31], schizophrenia [32], and Sjögren׳s syndrome [33] etc.

In this study, we also used a population-based database to investigate the demographic characteristics, the overall survival analysis and prescription patterns of individuals with hypertension among type 2 diabetes patients according to CHM usage. In addition, we also evaluate the effect of selected herbal formulas and single herbs on smooth muscle cell contractility.

## Results

### Demographic characteristics and overall survival analysis of individuals with hypertension among type 2 diabetes patients according to CHM usage

**Fig 1:**
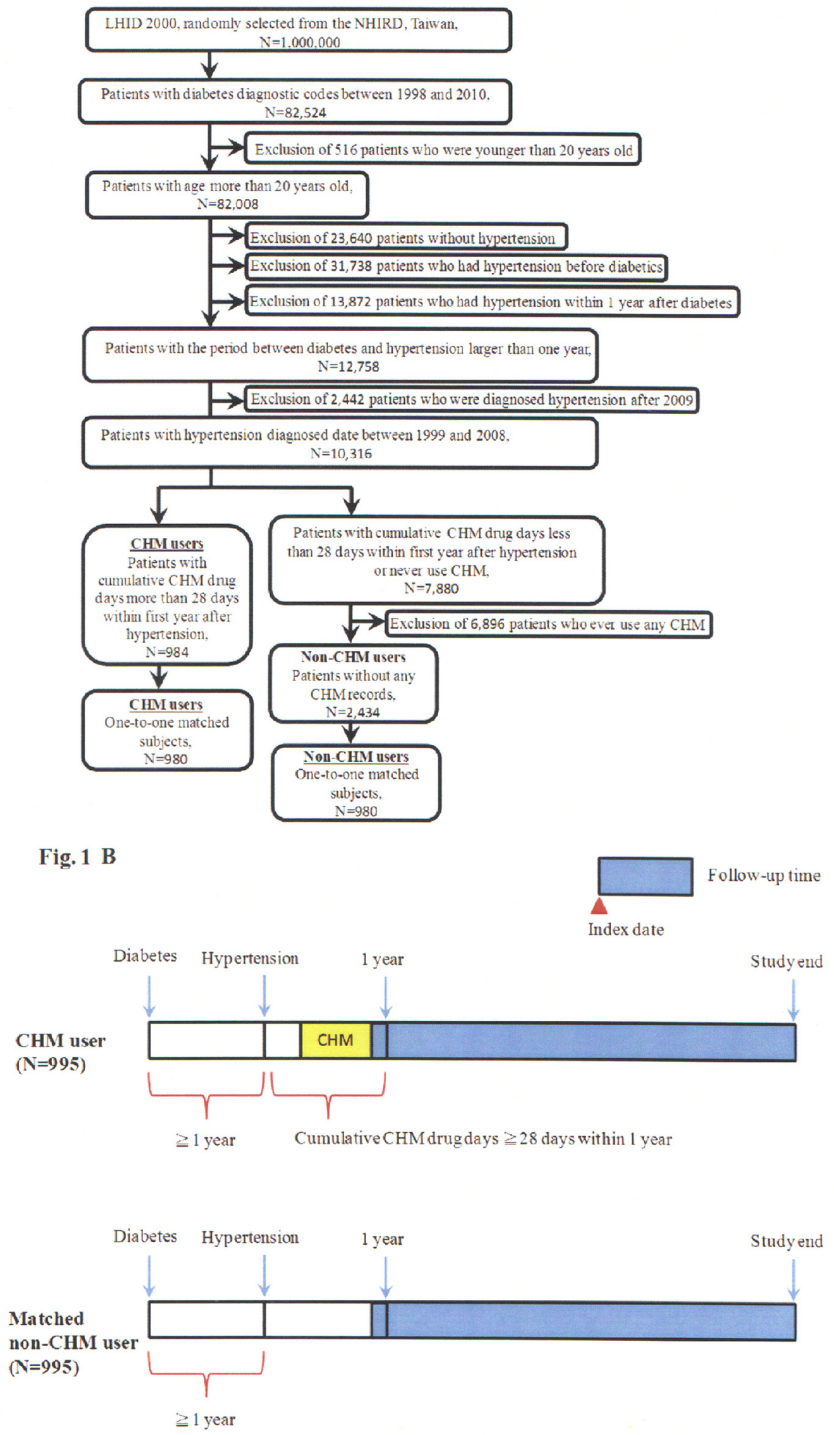
Flow recruitment diagram. **A:** Chart showing the protocol for enrollment of study subjects. **B:** Follow-up time for CHM and matched non-CHM users.

In this study, the database claims identified 984 CHM users and 2,434 non-CHM users with hypertension among type 2 diabetes patients from a cohort of one million randomly sampled cases from the National Health Insurance Research Database (NHIRD) [34] (Fig 1A). The demographic characteristics of CHM users versus non-CHM users (total subjects) are shown in the left side of Table 1. There were significant different frequency distributions for age, gender, duration from diabetes to hypertension, comorbidity (cardiovascular disease and hyperlipidaemia), and income for these two groups (*p* < 0.05). The CHM users (total subjects) were characterized with younger age, more females, longer duration time from diabetes to hypertension, lesser cases for cardiovascular disease, more cases for hyperlipidaemia, and higher incomes. The one-to-one match method was used to match CHM users and non-CHM users. After matching these two groups for age, gender and date of diagnosis of hypertension, CHM and non-CHM users were selected (Fig 1B and Table 1 right side). There were significant different frequency distributions for duration from diabetes to hypertension, and hyperlipidaemia (*p* < 0.05). The CHM users were characterized with slightly longer duration time from diabetes to hypertension, and more cases for hyperlipidaemia.

**Table 1:** Demographic characteristics of total subjects and frequency matched subjects with hypertension among type 2 diabetes patients according to CHM usage.

Characteristics	Total subjects						Frequency matched subjects					
	Total Number	non-CHM user		CHM user		*p* value	Total Number	non-CHM user		CHM user		*p* value
		N = 2,436		N = 984				N = 980		N = 980		
		N	%	N	%			N	%	N	%	
**Age**												
<60 yrs	1,406	965	39.61	441	44.82	***<0*.*0001***	902	465	47.45	437	44.59	0.6047
60~70 yrs	978	662	27.18	316	32.11		615	299	30.51	316	32.24	
70~80 yrs	767	584	23.97	183	18.6		361	178	18.16	183	18.67	
> = 80 yrs	269	225	9.24	44	4.47		82	38	3.88	44	4.49	
**Gender**												
Male	2,042	1,566	64.29	476	48.37	***<0*.*0001***	952	476	48.57	476	48.57	1
Female	1,378	870	35.71	508	51.63		1,008	504	51.43	504	51.43	
**Duration from diabetes to hypertension**												
1~2 years	919	694	28.49	225	22.87	***0*.*0006***	450	225	22.96	225	22.96	1
2~4 years	1512	1,074	44.09	438	44.51		874	437	44.59	437	44.59	
> = 5 years	989	668	27.42	321	32.62		636	318	32.45	318	32.45	
**Cardiovascular disease**												
No	2,852	1,999	82.06	853	86.69	***0*.*001***	1,680	831	84.8	849	86.63	0.2453
Yes	568	437	17.94	131	13.31		280	149	15.2	131	13.37	
**Ischaemic heart disease**												
No	2,664	1,889	77.55	775	78.76	0.4382	1,540	769	78.47	771	78.67	0.9123
Yes	756	547	22.45	209	21.24		420	211	21.53	209	21.33	
**Chronic kidney disease**												
No	3,225	2,288	93.92	937	95.22	0.1692	1,858	924	94.29	934	95.31	0.3092
Yes	195	148	6.08	47	4.78		102	56	5.71	46	4.69	
**Hyperlipidaemia**												
No	1,964	1,468	60.26	496	50.41	***<0*.*0001***	1,042	548	55.92	494	50.41	***0*.*0145***
Yes	1,456	968	39.74	488	49.59		918	432	44.08	486	49.59	
**Obesity**												
No	3,374	2,409	98.89	965	98.07	0.0587	1,927	966	98.57	961	98.06	0.38
Yes	46	27	1.11	19	1.93		33	14	1.43	19	1.94	
**Alcohol-related illness**												
No	3,369	2,397	98.4	972	98.78	0.4047	1,931	963	98.27	968	98.78	0.3496
Yes	51	39	1.6	12	1.22		29	17	1.73	12	1.22	
**Tobacco use**												
No	3,401	2,426	99.59	975	99.09	0.0726	1,948	977	99.69	971	99.08	0.0823
Yes	19	10	0.41	9	0.91		12	3	0.31	9	0.92	
**INCOME**												
<NT20000	739	582	23.89	157	15.96	***<0*.*0001***	359	202	20.61	157	16.02	***0*.*0072***
NT20000~NT30000	677	491	20.16	186	18.9		381	196	20	185	18.88	
NT30000~NT40000	1,438	982	40.31	456	46.34		894	441	45	453	46.22	
> = NT40000	566	381	15.64	185	18.8		326	141	14.39	185	18.88	
**Urbanization level**												
1	817	574	23.56	243	24.7	0.3331	488	245	25	243	24.8	0.4303
2	1,033	739	30.34	294	29.88		605	313	31.94	292	29.8	
3	497	340	13.96	157	15.96		290	133	13.57	157	16.02	
4	585	421	17.28	164	16.67		314	151	15.41	163	16.63	
5	488	362	14.86	126	12.8		263	138	14.08	125	12.76	

CHM, Chinese herbal medicine; N, number; NT, new Taiwan dollars.

Urbanization level: 1 indicates the hightest level of urbanization and 5 is the lowest level.

*p* values were obtained by chi-square test.

*p* value (*p* < 0.05) was highlighted in bold italic.

**Fig 2:**
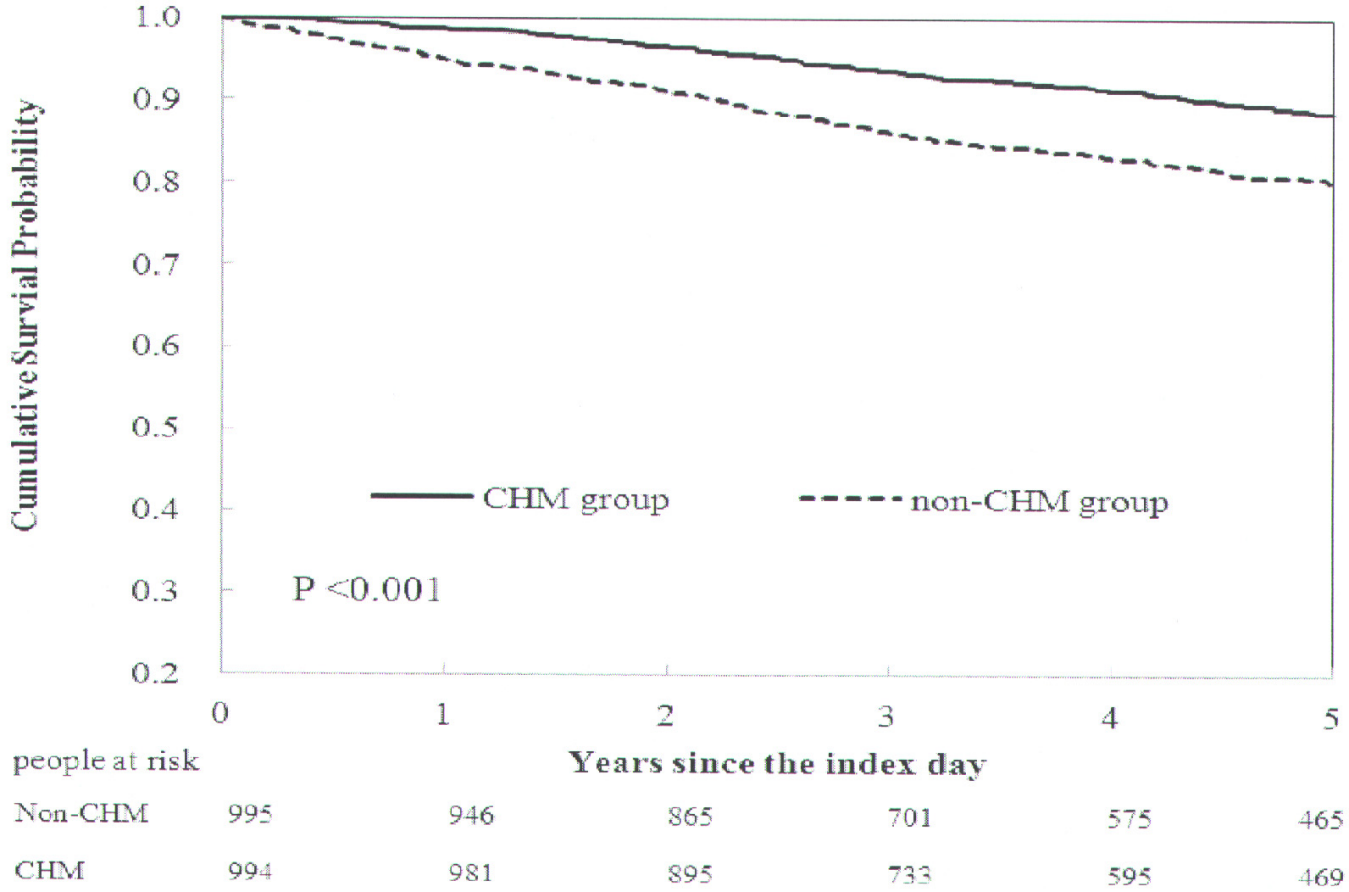
Cumulative survival curves of individuals with hypertension among type 2 diabetes patients according to Chinese herbal medicine (CHM) usage.

The cumulative survival probability of individuals with hypertension among type 2 diabetes patients according to CHM usage were shown in Fig 2. The overall survival rate was different between CHM users and matched non-CHM users (*p* < 0.001). The cumulative survival probabilities were higher in CHM users than in matched non-CHM users suggesting that CHM may be beneficial for longer survival of hypertension individuals among type 2 diabetes patients.

### Twelve most common herbal formulas and single herbs prescribed by TCM doctors for the treatment of hypertension individuals among type 2 diabetes patients

The 12 most common Chinese herbal formulas and single herbs prescribed for the CHM users analyzed in this study are listed in Table 2. The follow-up person-years were from hypertension to the study end as shown in Fig 1B. The herbal composition of these herbal formulas and single herbs were also shown in Table A in S1 File. Shu-Jing-Huo-Xue-Tang was the most commonly prescribed herbal formula, followed by Liu-Wei-Di-Huang-Wan and Jia-Wei-Xiao-Yao-San. Among the top 12 herbal formulas, Ji-Sheng-Shen-Qi-Wan and Zi-Bai-Di-Huang-Wan are 2 derivative formulas of Liu-Wei-Di-Huang-Wan. Therefore, Liu-Wei-Di-Huang-Wan and its various derivatives were the most common herbal formulas prescribed by Chinese medical doctors for the CHM users in this study. Of the 12 most common single herbs, Yan-Hu-Suo was the most commonly prescribed, followed by Dan-Shen and Ge-Gen.

**Table 2:** Twelve most common herbal formulas and single herbs prescribed by TCM doctors for the treatment of hypertension individuals among type 2 diabetes patients.

	Number of Person—years	Frequency of prescriptions	Percentage of usage person	Average daily dose (g)	Average duration for prescription (days)
**Total**	4,875	38,140	100	11.9	7.6
**Herbal formula**	4,858	36,685	99.6	9.1	7.6
Shu-Jing-Huo-Xue-Tang	2,153	1,995	39.2	3.9	6.8
Liu-Wei-Di-Huang-Wan	1,906	2,137	34.3	4	8.3
Jia-Wei-Xiao-Yao-San	1,719	1,625	32.4	4.1	8.5
Ge-Gen-Tang	1,674	1,190	31.2	4.3	6.7
Shao-Yao-Gan-Cao-Tang	1,643	1,150	30.7	3.3	7.2
Ma-Xing-Shi-Gan-Tang	1,549	1,349	29.1	3.9	6.2
Xue-Fu-Zhu-Yu-Tang	1,557	1,308	28.4	4	8.9
Du-Huo-Ji-Sheng-Tang	1,479	1,380	27.4	4.7	7.8
Chuan-Xiong-Cha-Tiao-San	1,404	1,062	26.9	4	6.2
Ji-Sheng-Shen-Qi-Wan	1,438	1,576	26	4.1	9.6
Gan-Lu-Yin	1,387	1,391	25.9	3.7	7.5
Zhi-Bai-Di-huang-Wan	1,437	1,364	25.8	4	10.1
**Single herb**	4,732	29,455	97.2	4	7.8
Yan-Hu-Suo	1,830	1,665	35.2	1.1	7.6
Ge-Gen	1,716	1,596	34.4	1.4	8.2
Dan-Shen	1,798	2,179	34.3	1.3	10.3
Tian-Hua-Fen	1,751	1,697	33.2	1.1	9.2
Jie-Geng	1,715	1,413	33	1	6.6
Bei-Mu	1,618	1,436	31.5	1.1	6.9
Huang-Qin	1,607	1,409	31.5	1.1	8
Niu-Xi	1,635	1,345	30.6	0.9	7.9
Mai-Men-Dong	1,516	1,287	28.6	1.2	8.9
Huang-Qi	1,473	1,807	28.3	1.4	8.8
Xuan-Shen	1,496	1,157	28.2	1.2	9
Xing-Ren	1,472	1,057	27.9	1.1	6.7

TCM, traditional Chinese medicine.

Follow-up time was from hypertension to the study end (Fig 1B).

### Effect of most common herbal formulas and single herbs on smooth muscle cell contractility

**Fig 3:**
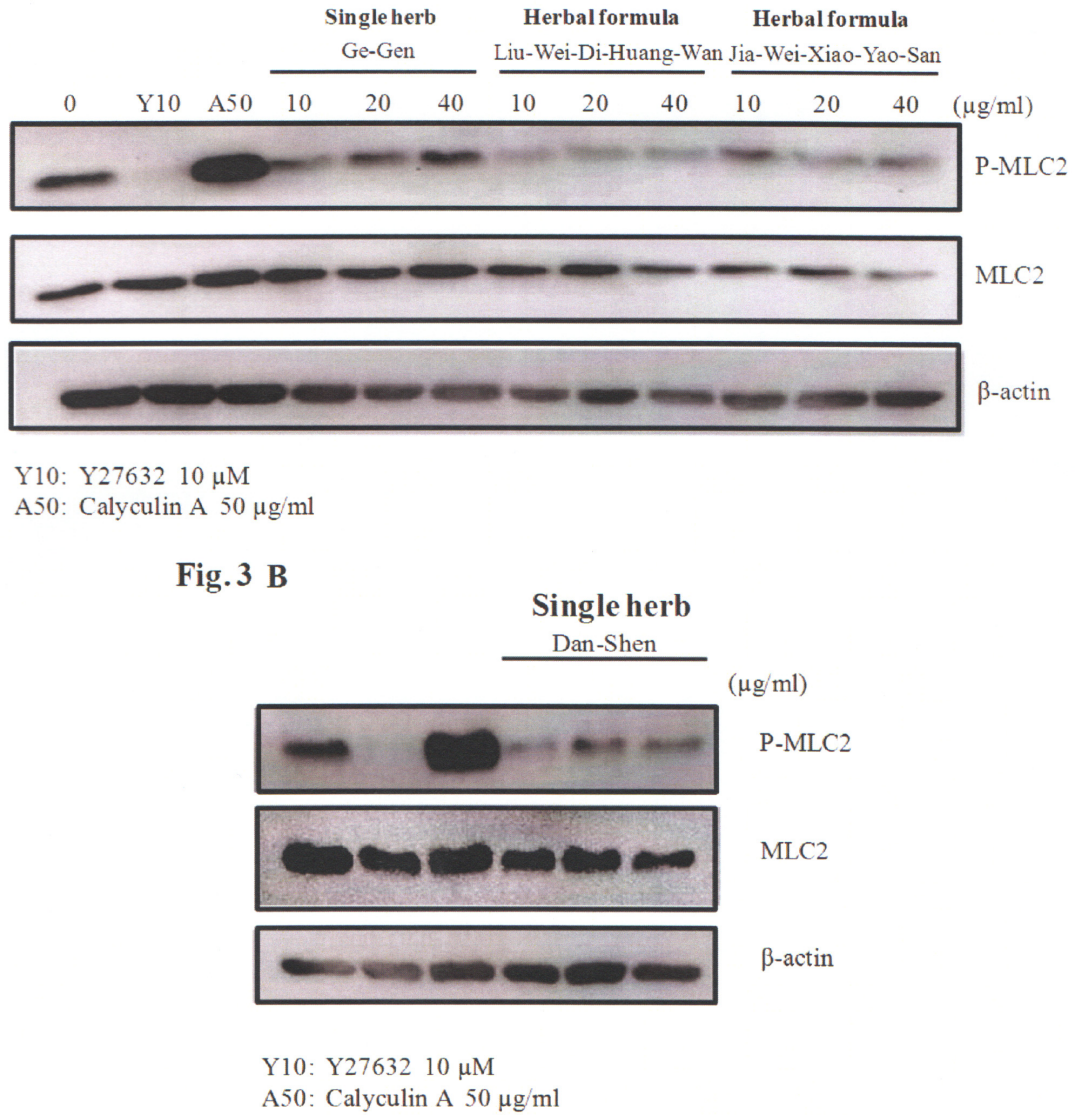
Effect of the four most common herbal formulas and single herbs on the phosphorylation of myosin light chain (MLC) protein. Briefly, A10 cells were treated with herbal formulas (A) or single herbs (B). Y27632 (Y10; 10 μM) and calyculin A (A50; 50 μg/ml) were used as negative and positive controls. Western blot analysis and staining with anti-phospho-MLC, anti-total-MLC, and anti-beta actin antibodies was then performed. Phospho-MLC, total-MLC, and beta actin were all obtained with their appropriate protein size bands. The relative Phospho-MLC intensity (%) was expressed as [(Phospho-MLC/total-MLC)_drug treated_/ (Phospho-MLC/total-MLC)_cell only_ x 100%]. The Mean±SEM values for at least three independent experiments along with the representative western blot were performed.

Smooth muscle cell contractility can be monitored by measuring the phosphorylation of myosin light chain protein [35]. The Y27632 compound (Rho kinase inhibitor) was used as the control for decreased myosin light chain phosphorylation [36]. And the calyculin compound was used as the control for increased myosin light chain phosphorylation [35]. We chose most common used two herbal formulas and two single herbs from these top 12 herbs (herbal formulas: Liu-Wei-Di-Huang-Wan and Jia-Wei-Xiao-Yao-San; single herbs: Dan-Shen and Ge-Gen) according to their frequencies of prescriptions and average duration for prescription. A10 cells (rat smooth muscle cells) were treated with these herbs at the concentrations as indicated (Fig 3A and 3B; S3A and S3B Fig). As shown, these four herbs reduced phosphorylation of myosin light chain. These herbs inhibited the phosphorylation of myosin light chain protein, suggesting that these most common herbs may be beneficial for smooth muscle cell contractility.

## Discussion

In this study, we used a population-based database to investigate the demographic characteristics, the overall survival analysis and prescription patterns of individuals with hypertension among type 2 diabetes patients according to CHM usage. In addition, we also evaluate the effect of two herbal formulas and two single herbs from these top 12 herbs on smooth muscle cell contractility. We found that the cumulative survival probabilities were higher in CHM users than in non-CHM users. We also described the most common prescribed CHMs. The single herbs and the herbal formulas inhibited smooth muscle cell contractility. Our results suggest that adjunctive CHM therapy treatment may improve the overall survival rate of individuals with hypertension among type 2 diabetes patients and some of them modulate smooth muscle cell contractility.

Our results showed that the overall survival rate was higher in CHM users than in non-CHM users from hypertension individuals among type 2 diabetes patients. And we also found that patients treated with any CHM, herbal formulas or single herbs had the trend of lower risks of the death, macrovascular and microvascular diseases as the endpoints after adjusted for age, duration from diabetes to hypertension, and comorbidities by the conditional logistic analysis (Tables B–G in S1 File).

The regular medical treatments (other than CHM) between CHM and non-CHM users showed that there were more patients in non-CHM users who have used anti-diabetes drugs from diabetes to the index date (Table H in S1 File). However, there were no anti-diabetes drug usage differences between these two groups from index date to index date + 365 (Table I in S1 File). As for the anti-hypertension drugs from diabetes to the index day (Table H in S1 File), there were more patients in non-CHM users who have used anti-hypertension drugs- ACEI or ARB (*p* < 0.05). However, there were more patients in CHM users who have used anti-hypertension drugs- beta blocking agents (*p* < 0.05). From index date to index date + 365 (Table I in S1 File), there were more patients in non-CHM users who have used anti-hypertension drugs- ACEI or ARB (*p* < 0.05). CHM has been reported to reduce progression from impaired glucose tolerance to diabetes [37, 38]. Furthermore, CHMs have been used to successfully treat diabetes via increased insulin secretion and sensitivity, enhanced glucose uptake by adipose and muscle tissues, inhibition of glucose absorption by the intestine, inhibition of glucose production by hepatocytes, and anti-inflammatory activity [21–23, 39].

The most common herbal formulas were Liu-Wei-Di-Huang-Wan and Jia-Wei-Xiao-Yao-San. Liu-Wei-Di-Huang-Wan were the most common herbal formula in this study. Its various derivatives (Ji-Sheng-Shen-Qi-Wan, and Zi-Bai-Di-Huang-Wan) were also noted in our herbal formula list. Liu-Wei-Di-Huang-Wan are composed of Rx. Rehmanniae Preparata, Fr. Corni, Rx. Dioscoreae, Poria, Cx. Moutan, and Rz. Alismatis. Liu-Wei-Di-Huang-Wan has been used to treat diabetes, pre-diabetes, fatigue, and metabolic syndrome [29, 40]. Furthermore, scientific evidence has suggested that Liu-Wei-Di-Huang-Wan can decrease visceral fat deposition [41], increase plasma levels of adiponectin and improve insulin resistance [42], and improve the lipid profile indicating a reduction of cardiovascular risk [43]. And Liu-Wei-Di-Huang-Wan combined with antihypertensive drugs appears to be effective in improving blood pressure and symptoms in patients with essential hypertension [44]. We have found that there were also no significant differences in the osmolarity and cell survival rate of cells among these herbal formulas and single herbs as compared with the cell only control, suggesting that the osmolarity of the Chinese herbal medicine are suitable for the cells in culture (S1A and S1B Fig). Furthermore, our functional analysis by measuring the phosphorylation of myosin light chain protein and the collage contraction assay (S2A–S2D Fig) first showed that smooth muscle cell contractility was inhibited by treatment with Liu-Wei-Di-Huang-Wan, which was in agreement with previous clinical observations [44]. Jia-Wei-Xiao-Yao-San are composed of Rx. Angelicae Sinensis, Rx. Paeoniae Alba, Poria, Rz. Atractylodis Macrocephalae, Rx. Bupleuri, Cx. Moutan, Fr. Gardeniae, and Rx. Gly. Jia-Wei-Xiao-Yao-San is used to treat symptoms including nervousness, palpitations, headache, anorexia, night sweating, dry eyes, hot flashes, and irregular menstruation; it also has hepatoprotective effects [45–49]. However, there were no related literatures related to the effect of Jia-Wei-Xiao-Yao-San on diabetes or hypertension. To our knowledge, this is the first study to show that Jia-Wei-Xiao-Yao-San can inhibit smooth muscle cell contractility by measuring the phosphorylation of myosin light chain protein and the collage contraction assay (S2A–S2D Fig).

Dan-Shen was the most common single herb and composed of Radix Salviae Miltiorrhizae. In previous studies, Dan-Shen has been shown to have protective effects on the cardiovascular system [50–54] and pulmonary arteries [55, 56]. Furthermore, active component (SalB) from Dan-Shen can exhibit antidiabetic activity and inhibit symptoms of diabetes mellitus in rats and these effects may partially be correlated with its insulin sensitivity, glycogen synthesis and antioxidant activities [57–60]. Ge-Gen was composed of Radix Puerariae and contains an isoflavonoid glycoside with hypotensive effects, with excellent clinical results in the treatment of hypertension [61]. Furthermore, puerarin is a major active ingredient of Ge-Gen and exerts significant protective effects against diabetic retinopathy in rats via regulating angiogenesis factors expressions [62]. Interestingly, our results suggest that patients treated with the single herb- Ge-Gen had the statistical significance of lower risk of acute myocardial infarction and nephropathy (Table B and F in S1 File). There were no trends or statistical significance observed from the ischemic stroke, hemorrhagic stroke and amputation as the endpoint (Table C–E in S1 File). We are the first to suggest that Dan-Shen and Ge-Gen were the most common single herbs for individuals with hypertension among type 2 diabetes patients and *in vitro* functional analysis suggested that smooth muscle cell contraction was inhibited by treatment with these herbs.

By integrating the National Health Insurance Research Database (NHIRD) review with our *in vitro* functional data, we were able to investigate the mechanism of action of CHM in the treatment of disease. Limitations of this study included a lack of blood physiological and biochemical measures in this database, such as blood pressure or blood sugar. The NHIRD limitations also include lacks of genetic factors, environmental factors (including levels of job stress and exercise), personal histories (including education and body mass index), and potential disease misclassifications [63–67]. The usage of CHM improves the overall survival rate of individuals with hypertension among type 2 diabetes patients and also these CHM treatment modulates smooth muscle cell contractility. Our study provides a CHM comprehensive list that may be useful in future investigation of the safety and efficacy for individuals with hypertension among type 2 diabetes patients.

## Materials and Methods

### Ethical statement

This study was evaluated and approved for the purchase of the National Health Insurance Research Database (NHIRD) by the Human Studies Committee of China Medical University Hospital, Taichung, Taiwan. No informed consent was required because the data were analyzed anonymously. The cell line rat aortic smooth muscle cell line A10 cells (BCRC number:60127; used in Fig 3A and 3B) were purchased from Food Industry Research and Development Institute in Taiwan (https://catalog.bcrc.firdi.org.tw/BSAS_cart/controller?event=SEARCH&bcrc_no=60127&type_id=4&keyword=smooth;;muscle;;cells). These cells were derived from the thoracic aorta of rats and served as a commonly used model of vascular smooth muscle cells [35] and were approved by the Animal Care and Use Committee (IACUC) of China Medical University, Taichung, Taiwan.

### National Health Insurance Research Database (NHIRD) resource in Taiwan

The national health insurance (NHI) program in Taiwan was started in 1995 to make health care available for all residents of Taiwan. As of 2010, over 99% of residents were enrolled in the program [68]. The NHI program provides the National Health Insurance Research Database (NHIRD) resource (http://nhird.nhri.org.tw/en/index.htm) for scientists in Taiwan and only for research purposes. Data for this study were retrieved from the “Longitudinal Health Insurance Database (LHID2000)”, which includes all the original claim data and registration files for 1,000,000 beneficiaries, randomly sampled from the year 2000 Registry for Beneficiaries (n = 23.72 million) under the NHI program. This database contains information on patient demographics, diagnoses, prescriptions, records of clinical visits and hospitalizations, inpatient orders, ambulatory care, and socio-demographic factors. Disease diagnoses are coded using the International Classification of Disease, 9^th^ Revision, Clinical Modification (ICD-9-CM). This database also include traditional Chinese medicine services (Chinese herbal medicine (CHM), acupuncture, and manipulative therapies; http://www.nhi.gov.tw/English/webdata/webdata.aspx?menu=11&menu_id=592&WD_ID=592&webdata_id=3161). The data are from the National Health Insurance Research Database, Taiwan (NHIRD) http://nhird.nhri.org.tw/en/index.html. Contact nhird@nhri.org.tw for details and data access.

### Study population

This study was designed as a population-based retrospective cohort study. In this study, a cohort of one million individuals randomized selected from NHI (Taiwan) was used. The sampled population was representative of all NHI beneficiaries. The study subjects were selected from this cohort and were shown in Fig 1A. There were 84,032 individuals with diabetes (ICD-9-CM: 250) between 1998 and 2010. The hypertension ICD-9-CM used in this study was from 401–405. Individuals under the age of 20 were excluded. Individuals without hypertension (ICD-9-CM: 401–405), who had hypertension before diabetes, who had hypertension within 1 year after diabetes, and who had hypertension after 2009 were also excluded. In addition, at least one of the following enrollment criteria had to med for identifying patients with hypertension in the study: (1) one or more inpatient admissions with diagnosis of hypertension, or (2) three or more outpatients visits within one-year period, each with a diagnosis of hypertension. The first date which satisfied the above (1) or (2) criteria was defined as the date of diagnosis of hypertension. After all of these criteria were applied, 10,664 study subjects were included in the study cohort.

### Definition of CHM and non-CHM users

Study subjects with a record of cumulative CHM drug days more than 28 within first year after hypertension were defined as CHM users (N = 984, Fig 1B). Study subjects with no recorded of CHM usage were defined as non-CHM users (N = 2,434). The date of satisfying the criterion of cumulative 28 drug days of CHM prescription was designated as the index date (Fig 1B). The one-to-one match method was used to match CHM users and non-CHM users. After matching these two groups for age, gender and date of diagnosis of hypertension, CHM and non-CHM users were selected (Fig 1B and Table 1 right side). A total of 980 subjects for each group were selected. The study endpoint was as the following: date of death, date of withdrawal from the NHI program, or date of follow-up termination (31 Dec. 2010). This study was designed as a population-based retrospective cohort study and to explore the effect of Chinese Herbal Medicine treatment on the overall survival rate of individuals with hypertension among type 2 diabetes patients.

### Chinese herbal medicine (CHM)

All drug codes for CHM (herbal formulas and single herbs) were collected and grouped according to their name. The frequencies of prescriptions, cumulative drug doses, average durations of per prescription, and follow-up person years were calculated from hypertension to the study end for the CHM users. Herbal formulas usually constituted a combination of 2 to 17 herbs (Table A in S1 File), created by experienced TCM doctors; these formulas have been used for thousands of years. Single herbs were obtained from plant, animal, or mineral material and can be mixed with other herbs to create a formula. The herbal formulas and single herbs in the NHIRD database were all produced by Good Manufacturing Practice (GMP) certified traditional Chinese medicine manufacturers based in Taiwan. These manufacturers included Sun Ten Pharmaceutical Co. Ltd., Shang Chang Pharmaceutical Co. Ltd., Chuang Song Zong Pharmaceutical Co. Ltd., KO DA Pharmaceutical Co. Ltd., and Kaiser Pharmaceutical Co. Ltd.

### Study covariates

We collected demographic data such as age, gender, income and urbanization levels. Urbanization levels in Taiwan are divided into five strata according to the Taiwan National Health Research Institute publications, with level 1 referring to the most urbanized communities and level 5 referring to the least urbanized communities. We identified the diagnoses of comorbidities which defined by following diagnoses recorded before the diagnosis date of hypertension: cardiovascular disease (ICD-9-CM: 430–437), ischaemic heart disease (ICD-9-CM: 410–414), chronic kidney disease (ICD-9-CM: 582–583) and hyperlipidaemia (ICD-9-CM: 272) (Table 1). The diagnosis criteria for each comorbidity were similar to those for hypertension.

We also applied the conditional multivariable logistic regression adjusted for all variables in the Table 1 to assess the effect of CHM on the occurrence of blood pressure related disease such as acute myocardial infarction, ischemic stroke, hemorrhagic stroke, amputation, and nephropathy (the results were shown in Tables B–F in S1 File). The covariates included CHM user, age, income, duration from diabetes to hypertension, and comorbidities before hypertension including cardiovascular disease (ICD-9-CM: 430–437), ischaemic heart disease (ICD-9-CM: 410–414), chronic kidney disease (ICD-9-CM: 582–583) and hyperlipidaemia (ICD-9-CM: 272).

### Cell culture, reagents, and Western blotting

Rat aortic smooth muscle cells (A10 cell line) were cultured in Dulbecco's Modified Eagle's Medium (DMEM) supplemented with 10% fetal bovine serum (FBS), 100 U/mL penicillin, 100 U/mL streptomycin, and 2 mM L-glutamine (Gibco). Y27632, and calyculin A were purchased from Sigma (St. Louis, MO, USA). A10 cells were treated with Y27632 (10 μM), and calyculin A (50 μg/ml) for 10 min. The treated cells were lysed in RIPA buffer (Thermo Scientific ^™^) and then were applied to Western blot analysis and staining with anti-phospho-myosin light chain (MLC) (1:1,000 dilution), anti-total-MLC (1:1,000 dilution), and anti-beta actin (1:1,000 dilution) antibodies (Fig 3; S3 Fig). The monoclonal anti-phospho-MLC (phospho-myosin light chain 2 [Ser19] mouse mAb; catalog number: 3675) and polyclonal anti-total-MLC (myosin light chain 2 antibody; catalog number: 3672) rabbit antibodies were from Cell Signaling Technology, Inc. The anti-beta actin (actin antibody [mAbGEa]; catalog number: NB100-74340) mouse monoclonal antibody was obtained from Novus Biologicals. The experimental protocol used for Western blotting has been described previously [69, 70]. Briefly, cells were harvested, washed, and lysed in lysis buffer (50 mM Tris-HCl [pH 7.5], 150 mM NaCl, 5 mM EDTA, 1% Triton X-100, 0.1% SDS) supplemented with protease inhibitor cocktail (Roche). The lysates were resolved by 12% SDS-PAGE and transferred to polyvinylidene fluoride membranes (Millipore). The membranes were incubated with primary antibodies overnight at 4°C and then incubated with alkaline phosphatase-conjugated secondary antibodies (Goat anti-Mouse IgG (H+L) Polyclonal Secondary Antibody, HRP conjugate; 1:5000 dilutions; catalog number: A16072; Thermo Fisher Scientific). Signals were visualized using a SuperSignal West Femto Maximum Sensitivity Substrate Detection Kit; catalog number: 34096; Thermo Fisher Scientific) in accordance with the manufacturer's instructions.

### Statistical analysis

We presented demographic data such as age, gender, duration from diabetes to hypertension, comorbidities (cardiovascular disease, ischaemic heart disease, chronic kidney disease, and hyperlipidaemia), income, and urbanization level for both groups (CHM and non-CHM users) using count and percentage for categorical variables, and used chi-squared tests to assess their differences (Table 1). We sorted the cumulative person-years for each herbal formula and single herb and listed the top 12 most common herbal formulas and single herbs (Table 2). We employed Kaplan-Meier method to estimate the cumulative survival probabilities and used the log-rank test to explore the effect of Chinese Herbal Medicine treatment on the overall survival rate of individuals with hypertension among type 2 diabetes patients (CHM and non-CHM users; Fig 2). We also used conditional logistic analysis to explore the effect of CHM therapy as well as these most commonly used herbs on the reduction of macrovascular and microvascular diseases, and death as the endpoints during follow up (Tables B–G in S1 File). All *p*-values less than 0.05 were considered significant. All data management and statistical analyses were performed using Statistical Analysis System (SAS) software (version 9.3; SAS Institute, Cary, NC, USA).

## Supporting Information

S1 Fig Osmolarity and cell survival rate of cells treated with Chinese herbal medicine. **(A)** Detection of the osmolarity from the cell culture medium as shown above by using Vapro TM Osmometer, Model 5520. The standard 290 and the concentrations of NaCl (0%, 0.5%, 1%, and 2%) were used as the controls. Cells were in the presence of cells only, Y10 (Y27632 at 10 μM), single herbs- Dan-Shen and Ge-Gen, and herbal formula- Liu-Wei-Di-Huang-Wan and Jia-Wei-Xiao-Yao-San (5 and 10 μg/ml). Similar results were obtained in three independent experiments. Values represent the mean± S.D. **(B)** % of cell survival rate of cells treated with Chinese herbal medicine. Cells were in the presence of cells only, Y10 (Y27632 at 10 μM), single herbs- Dan-Shen and Ge-Gen, and herbal formula- Liu-Wei-Di-Huang-Wan and Jia-Wei-Xiao-Yao-San (5 and 10 μg/ml) for 24 h and were detected by using the WST-1 assay. Similar results were obtained in three independent experiments. Values represent the mean± S.D.
(PPTX)

S2 Fig Effect of Chinese herbs on contraction of collagen gels. Cell-embedded collagen gels were prepared according to the manufacturer‘s instructions (CELL BIOLABS, INC., cell contraction assay (catalog number CBA-201-T). (**A)** The surface area of collagen gels was calculated at 24 h, 48 h, and 120 h in the presence of cells only, Y10 (Y27632 at 10 μM), 1X BDM-contraction inhibitor as the controls. The surface area of collagen gels of single herbs- Dan-Shen and Ge-Gen, and herbal formula- Liu-Wei-Di-Huang-Wan and Jia-Wei-Xiao-Yao-San (5 and 10 μg/ml) was also calculated. The contraction of collagen gel was expressed in a percentage, with the surface area of the cells only serving as 100%. Similar results were obtained in three independent experiments. Values represent the mean± S.D. (**B)** The surface area of collagen gels was shown at 24 h in the presence of cells only (No. 1), Y10 (No. 2; Y27632 at 10 μM), 1X BDM-contraction inhibitor (No. 3) as the controls. The surface area of collagen gels of single herbs- Dan-Shen (No. 4; 5 μg/ml) and Ge-Gen (No. 5; 5 μg/ml), and herbal formula- Liu-Wei-Di-Huang-Wan (No. 6; 5 μg/ml) and Jia-Wei-Xiao-Yao-San (No. 7; 5 μg/ml) was also shown. (**C)** The surface area of collagen gels was shown at 48 h in the presence of cells only (No. 1), Y10 (No. 2; Y27632 at 10 μM), 1X BDM-contraction inhibitor (No. 3) as the controls. The surface area of collagen gels of single herbs- Dan-Shen (No. 4; 5 μg/ml) and Ge-Gen (No. 5; 5 μg/ml), and herbal formula- Liu-Wei-Di-Huang-Wan (No. 6; 5 μg/ml) and Jia-Wei-Xiao-Yao-San (No. 7; 5 μg/ml) was also shown. (**D)** The surface area of collagen gels was shown at 120 h in the presence of cells only (No. 1), Y10 (No. 2; Y27632 at 10 μM), 1X BDM-contraction inhibitor (No. 3) as the controls. The surface area of collagen gels of single herbs- Dan-Shen (No. 4; 5 μg/ml) and Ge-Gen (No. 5; 5 μg/ml), and herbal formula- Liu-Wei-Di-Huang-Wan (No. 6; 5 μg/ml) and Jia-Wei-Xiao-Yao-San (No. 7; 5 μg/ml) was also shown.
(PPTX)

S3 Fig Original uncropped and unadjusted blots of Fig 3. **(A)** herbal formulas; (**B)** single herbs. The antibodies (anti-Phospho-MLC, anti- Total-MLC, and anti-β-actin) used here were shown in the left of the S3 Fig.
(PPTX)

S1 File Supporting tables for Chinese herbal medicine treatment in hypertension individuals among type 2 diabetes patients. Herbal composition of twelve most common herbal formulas and single herbs prescribed by TCM doctors for the treatment of hypertension individuals among type 2 diabetes patients (Table A). Results of conditional multivariable logistic regression on the occurrence of acute myocardial infarction (Table B). Results of conditional multivariable logistic regression on the occurrence of ischemic stroke (Table C). Results of conditional multivariable logistic regression on the occurrence of hemorrhagic stroke (Table D).Results of conditional multivariable logistic regression on the occurrence of amputation (Table E).Results of conditional multivariable logistic regression on the occurrence of nephropathy (Table F).Results of conditional multivariable logistic regression on the occurrence of death (Table G).Regular medical treatment (from diabetes to index day) among type 2 diabetes patients according to CHM usage (Table H).Regular medical treatment (from index day to index day +365) among type 2 diabetes patients according to CHM usage (Table I).
(DOCX)
