# Supplementary material for: Chinese Herbal Medicine Treatment Improves the Overall Survival Rate of Individuals with Hypertension among Type 2 Diabetes Patients and Modulates In Vitro Smooth Muscle Cell Contractility
Source: PLoS One. 2015 Dec 23;10(12):e0145109. doi: 10.1371/journal.pone.0145109 (PMC4689379; doi:10.1371/journal.pone.0145109)
Supplement: S1 Fig — (A) Detection of the osmolarity from the cell culture medium as shown above by using Vapro TM Osmometer, Model 5520. The standard 290 and the concentrations of NaCl (0%, 0.5%, 1%, and 2%) were used as the controls. Cells were in the presence of cells only, Y10 (Y27632 at 10 μM), single herbs- Dan-Shen and Ge-Gen, and herbal formula- Liu-Wei-Di-Huang-Wan and Jia-Wei-Xiao-Yao-San (5 and 10 μg/ml). Similar results were obtained in three independent experiments. Values represent the mean± S.D. (B) % of cell survival rate of cells treated with Chinese herbal medicine. Cells were in the presence of cells only, Y10 (Y27632 at 10 μM), single herbs- Dan-Shen and Ge-Gen, and herbal formula- Liu-Wei-Di-Huang-Wan and Jia-Wei-Xiao-Yao-San (5 and 10 μg/ml) for 24 h and were detected by using the WST-1 assay. Similar results were obtained in three independent experiments. Values represent the mean± S.D. (PPTX) [file pone.0145109.s001.pptx]

## Slide 1
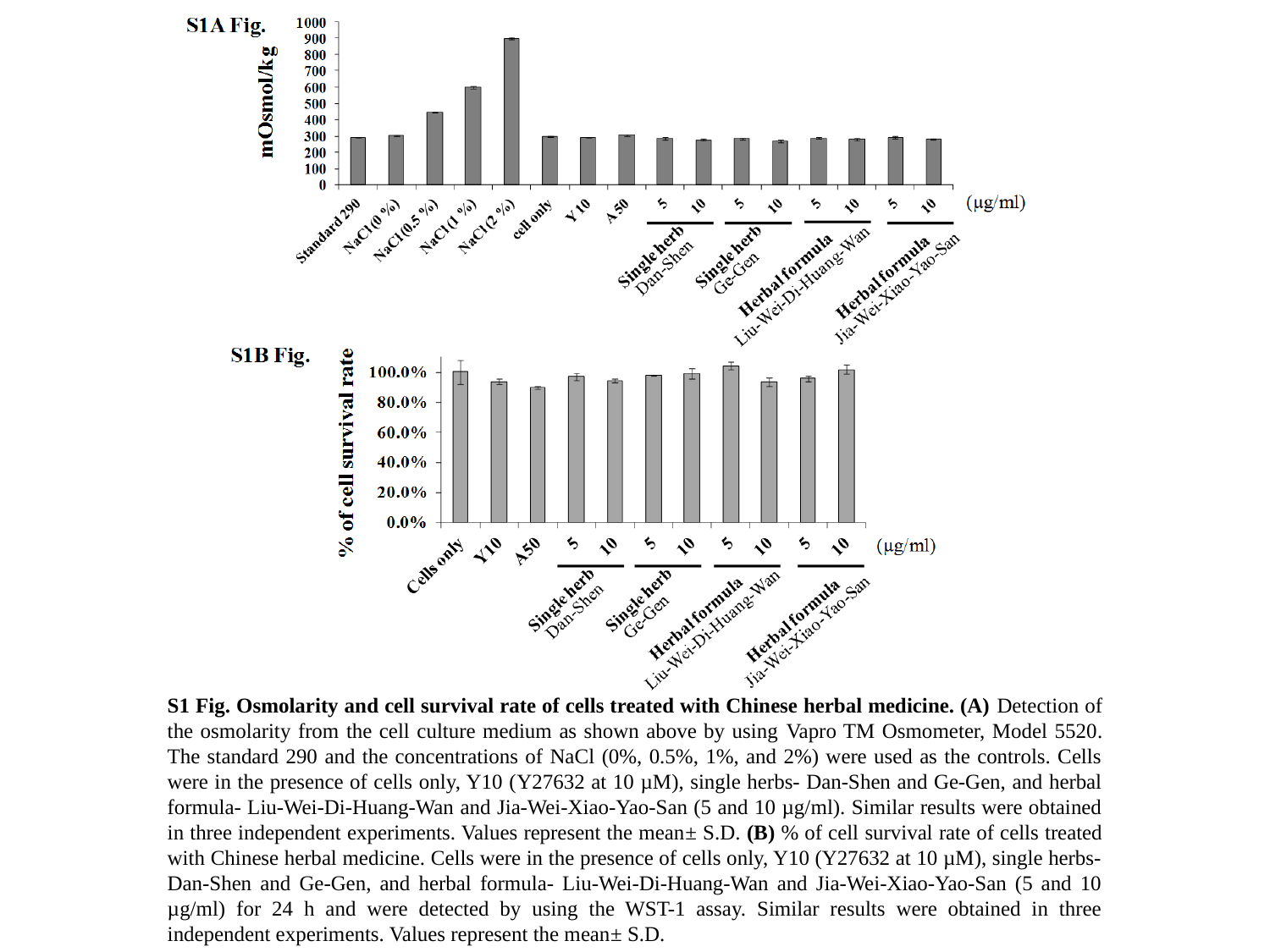

S1 Fig. Osmolarity and cell survival rate of cells treated with Chinese herbal medicine. (A) Detection of the osmolarity from the cell culture medium as shown above by using Vapro TM Osmometer, Model 5520. The standard 290 and the concentrations of NaCl (0%, 0.5%, 1%, and 2%) were used as the controls. Cells were in the presence of cells only, Y10 (Y27632 at 10 µM), single herbs- Dan-Shen and Ge-Gen, and herbal formula- Liu-Wei-Di-Huang-Wan and Jia-Wei-Xiao-Yao-San (5 and 10 µg/ml). Similar results were obtained in three independent experiments. Values represent the mean± S.D. (B) % of cell survival rate of cells treated with Chinese herbal medicine. Cells were in the presence of cells only, Y10 (Y27632 at 10 µM), single herbs- Dan-Shen and Ge-Gen, and herbal formula- Liu-Wei-Di-Huang-Wan and Jia-Wei-Xiao-Yao-San (5 and 10 µg/ml) for 24 h and were detected by using the WST-1 assay. Similar results were obtained in three independent experiments. Values represent the mean± S.D.
