# Supplementary material for: Chinese Herbal Medicine Treatment Improves the Overall Survival Rate of Individuals with Hypertension among Type 2 Diabetes Patients and Modulates In Vitro Smooth Muscle Cell Contractility
Source: PLoS One. 2015 Dec 23;10(12):e0145109. doi: 10.1371/journal.pone.0145109 (PMC4689379; doi:10.1371/journal.pone.0145109)
Supplement: S2 Fig — Cell-embedded collagen gels were prepared according to the manufacturer‘s instructions (CELL BIOLABS, INC., cell contraction assay (catalog number CBA-201-T). (A) The surface area of collagen gels was calculated at 24 h, 48 h, and 120 h in the presence of cells only, Y10 (Y27632 at 10 μM), 1X BDM-contraction inhibitor as the controls. The surface area of collagen gels of single herbs- Dan-Shen and Ge-Gen, and herbal formula- Liu-Wei-Di-Huang-Wan and Jia-Wei-Xiao-Yao-San (5 and 10 μg/ml) was also calculated. The contraction of collagen gel was expressed in a percentage, with the surface area of the cells only serving as 100%. Similar results were obtained in three independent experiments. Values represent the mean± S.D. (B) The surface area of collagen gels was shown at 24 h in the presence of cells only (No. 1), Y10 (No. 2; Y27632 at 10 μM), 1X BDM-contraction inhibitor (No. 3) as the controls. The surface area of collagen gels of single herbs- Dan-Shen (No. 4; 5 μg/ml) and Ge-Gen (No. 5; 5 μg/ml), and herbal formula- Liu-Wei-Di-Huang-Wan (No. 6; 5 μg/ml) and Jia-Wei-Xiao-Yao-San (No. 7; 5 μg/ml) was also shown. (C) The surface area of collagen gels was shown at 48 h in the presence of cells only (No. 1), Y10 (No. 2; Y27632 at 10 μM), 1X BDM-contraction inhibitor (No. 3) as the controls. The surface area of collagen gels of single herbs- Dan-Shen (No. 4; 5 μg/ml) and Ge-Gen (No. 5; 5 μg/ml), and herbal formula- Liu-Wei-Di-Huang-Wan (No. 6; 5 μg/ml) and Jia-Wei-Xiao-Yao-San (No. 7; 5 μg/ml) was also shown. (D) The surface area of collagen gels was shown at 120 h in the presence of cells only (No. 1), Y10 (No. 2; Y27632 at 10 μM), 1X BDM-contraction inhibitor (No. 3) as the controls. The surface area of collagen gels of single herbs- Dan-Shen (No. 4; 5 μg/ml) and Ge-Gen (No. 5; 5 μg/ml), and herbal formula- Liu-Wei-Di-Huang-Wan (No. 6; 5 μg/ml) and Jia-Wei-Xiao-Yao-San (No. 7; 5 μg/ml) was also shown. (PPTX) [file pone.0145109.s002.pptx]

## Slide 1
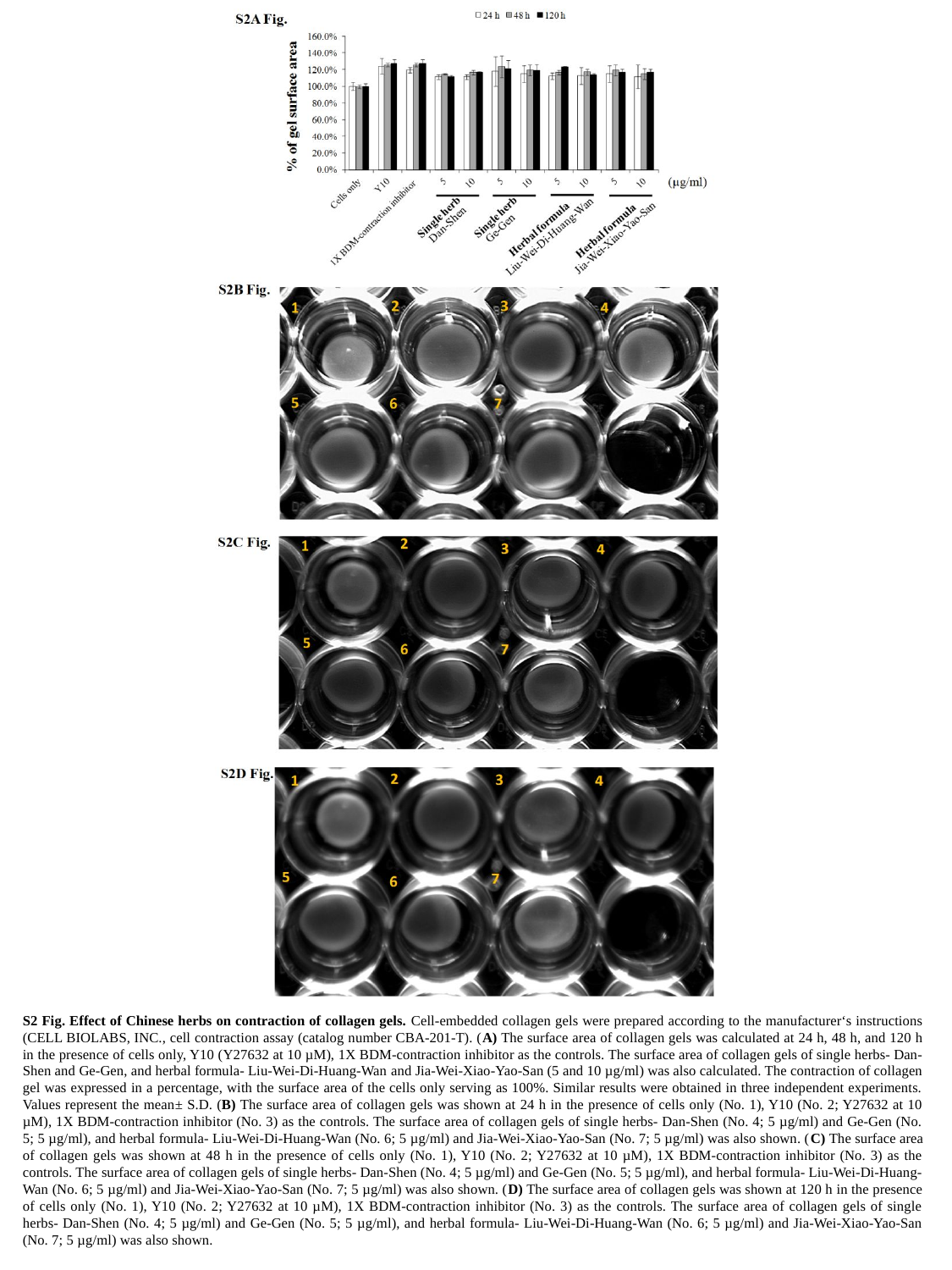

S2 Fig. Effect of Chinese herbs on contraction of collagen gels. Cell-embedded collagen gels were prepared according to the manufacturer‘s instructions (CELL BIOLABS, INC., cell contraction assay (catalog number CBA-201-T). (A) The surface area of collagen gels was calculated at 24 h, 48 h, and 120 h in the presence of cells only, Y10 (Y27632 at 10 µM), 1X BDM-contraction inhibitor as the controls. The surface area of collagen gels of single herbs- Dan-Shen and Ge-Gen, and herbal formula- Liu-Wei-Di-Huang-Wan and Jia-Wei-Xiao-Yao-San (5 and 10 µg/ml) was also calculated. The contraction of collagen gel was expressed in a percentage, with the surface area of the cells only serving as 100%. Similar results were obtained in three independent experiments. Values represent the mean± S.D. (B) The surface area of collagen gels was shown at 24 h in the presence of cells only (No. 1), Y10 (No. 2; Y27632 at 10 µM), 1X BDM-contraction inhibitor (No. 3) as the controls. The surface area of collagen gels of single herbs- Dan-Shen (No. 4; 5 µg/ml) and Ge-Gen (No. 5; 5 µg/ml), and herbal formula- Liu-Wei-Di-Huang-Wan (No. 6; 5 µg/ml) and Jia-Wei-Xiao-Yao-San (No. 7; 5 µg/ml) was also shown. (C) The surface area of collagen gels was shown at 48 h in the presence of cells only (No. 1), Y10 (No. 2; Y27632 at 10 µM), 1X BDM-contraction inhibitor (No. 3) as the controls. The surface area of collagen gels of single herbs- Dan-Shen (No. 4; 5 µg/ml) and Ge-Gen (No. 5; 5 µg/ml), and herbal formula- Liu-Wei-Di-Huang-Wan (No. 6; 5 µg/ml) and Jia-Wei-Xiao-Yao-San (No. 7; 5 µg/ml) was also shown. (D) The surface area of collagen gels was shown at 120 h in the presence of cells only (No. 1), Y10 (No. 2; Y27632 at 10 µM), 1X BDM-contraction inhibitor (No. 3) as the controls. The surface area of collagen gels of single herbs- Dan-Shen (No. 4; 5 µg/ml) and Ge-Gen (No. 5; 5 µg/ml), and herbal formula- Liu-Wei-Di-Huang-Wan (No. 6; 5 µg/ml) and Jia-Wei-Xiao-Yao-San (No. 7; 5 µg/ml) was also shown.
