# Supplementary material for: Chinese Herbal Medicine Treatment Improves the Overall Survival Rate of Individuals with Hypertension among Type 2 Diabetes Patients and Modulates In Vitro Smooth Muscle Cell Contractility
Source: PLoS One. 2015 Dec 23;10(12):e0145109. doi: 10.1371/journal.pone.0145109 (PMC4689379; doi:10.1371/journal.pone.0145109)
Supplement: S3 Fig — (A) herbal formulas; (B) single herbs. The antibodies (anti-Phospho-MLC, anti- Total-MLC, and anti-β-actin) used here were shown in the left of the S3 Fig. (PPTX) [file pone.0145109.s003.pptx]

## Slide 1
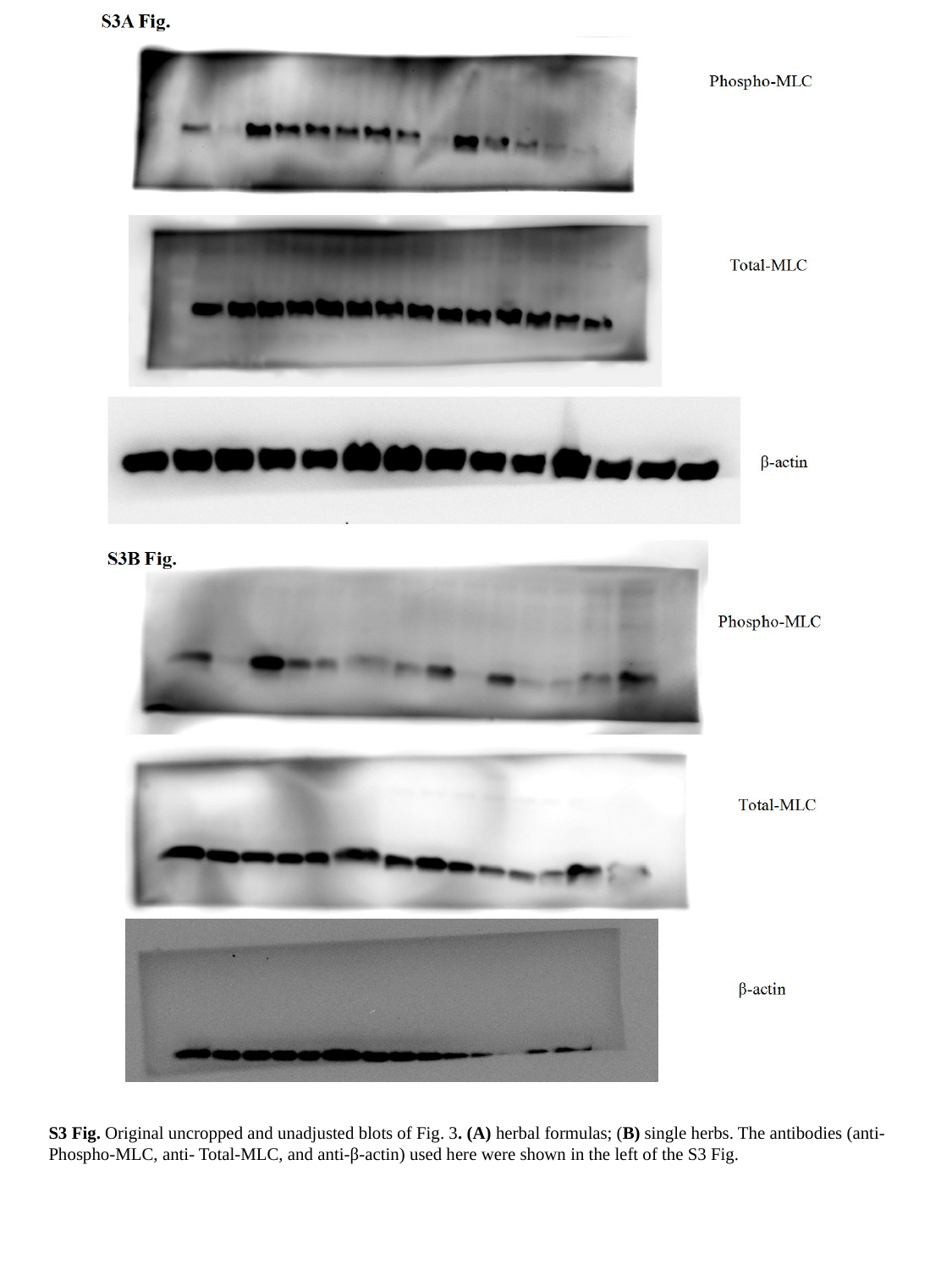

S3 Fig. Original uncropped and unadjusted blots of Fig. 3. (A) herbal formulas; (B) single herbs. The antibodies (anti-Phospho-MLC, anti- Total-MLC, and anti-β-actin) used here were shown in the left of the S3 Fig.
