# Supplementary material for: Chinese Herbal Medicine Treatment Improves the Overall Survival Rate of Individuals with Hypertension among Type 2 Diabetes Patients and Modulates In Vitro Smooth Muscle Cell Contractility
Source: PLoS One. 2015 Dec 23;10(12):e0145109. doi: 10.1371/journal.pone.0145109 (PMC4689379; doi:10.1371/journal.pone.0145109)
Supplement: S1 File — Herbal composition of twelve most common herbal formulas and single herbs prescribed by TCM doctors for the treatment of hypertension individuals among type 2 diabetes patients (Table A). Results of conditional multivariable logistic regression on the occurrence of acute myocardial infarction (Table B). Results of conditional multivariable logistic regression on the occurrence of ischemic stroke (Table C). Results of conditional multivariable logistic regression on the occurrence of hemorrhagic stroke (Table D).Results of conditional multivariable logistic regression on the occurrence of amputation (Table E).Results of conditional multivariable logistic regression on the occurrence of nephropathy (Table F).Results of conditional multivariable logistic regression on the occurrence of death (Table G).Regular medical treatment (from diabetes to index day) among type 2 diabetes patients according to CHM usage (Table H).Regular medical treatment (from index day to index day +365) among type 2 diabetes patients according to CHM usage (Table I). (DOCX) [file pone.0145109.s004.docx]

| **Table A in S1 File. Herbal composition of twelve most common herbal formulas and single herbs prescribed by TCM doctors for the treatment of hypertension individuals among type 2 diabetes patients** | | |
| --- | --- | --- |
| **CHM prescription** | **Number of herbs** | **Composition (Latin name)** |
| **Herbal formulas^a^** |  |  |
| Shu-Jing-Huo-Xue-Tang | 17 | Rx. Paeoniae Alba, Rx. Angelicae Sinensis, Rz. Chuanxiong, Rx. Rehmanniae, Sm. Persicae, Rz. Atractylodis, Poria, Rx. Achyranthis Bidentatae, Rx. Clematidis, Rx. Stephaniae Tetrandrae, Rz. seu Rx. Notopterygii, Rx. Saposhnikoviae, Rx. Gentianae, Rx. Angelicae Dahuricae, Per. Citri Reticulatae, Rx. Glycyrrhizae, Rz. Zingiberis Recens |
| Liu-Wei-Di-Huang-Wan | 6 | Rx. Rehmanniae Preparata, Fr. Corni, Rx. Dioscoreae, Poria, Cx. Moutan, Rz. Alismatis |
| Jia-Wei-Xiao-Yao-San | 10 | Rx. Angelicae Sinensis, Rx. Paeoniae Alba, Poria, Rz. Atractylodis Macrocephalae, Rx. Bupleuri, Cx. Moutan, Fr. Gardeniae, Rx. Glycyrrhizae Preparata, Hb. Menthae Haplocalycis, Rz. Zingiberis Recens |
| Ge-Gen-Tang | 7 | Rx. Puerariae, Hb. Ephedrae, Ram. Cinnamomi, Rx. Paeoniae Alba, Rz. Zingiberis Recens, Fr. Jujube, Rx. Glycyrrhizae |
| Shao-Yao-Gan-Cao-Tang | 2 | Rx. Paeoniae Alba, Rx. Glycyrrhizae Preparata |
| Ma-Xing-Shi-Gan-Tang | 4 | Hb. Ephedrae, Sm. Armeniacae, Gypsum Fibrosum, Rx. Glycyrrhizae Preparata |
| Xue-Fu-Zhu-Yu-Tang | 11 | Sm. Persicae, Flos Carthami, Rx. Angelicae Sinensis, Rz. Chuanxiong, Rx. Paeoniae Rubra, Rx. Cyathulae (or Rx. Achyranthis Bidentatae), Rx. Bupleuri, Rx. Platycodi, Fr. Aurantii, Rx. Rehmanniae, Rx. Glycyrrhizae |
| Du-Huo-Ji-Sheng-Tang | 15 | Rx. Angelica Pubescentis, Hb. Asari, Rx. Saposhnikoviae, Rx. Gentianae Macrophyllae, Hb. Taxilli, Cx. Eucommiae, Rx. Achyranthis Bidentatae, Cx. Cinnamomi, Rx. Angelica Sinensis, Rx. Chuanxiong, Rx. Rehmanniae, Rx. Paeoniae Alba, Rx. Ginseng, Poriae, Rx. Glycyrrhizae Preparata |
| Chuan-Xiong-Cha-Tiao-San | 9 | Hb. Menthae Haplocalycis, Rz. Chuanxiong, Rx. Angelicae Dahuricae, Rz. seu Rx. Notopterygii, Hb. Asari (or Dry-fried Rz. Cyperi), Hb. Schizonepetae, Rx. Saposhnikoviae, Rx. Glycyrrhizae Preparata, Fol. Camelliae |
| Ji-Sheng-Shen-Qi-Wan | 10 | Rx. Rehmanniae Preparata, Fr. Corni, Rx. Dioscoreae, Rz. Alismatis, Poriae, Cx. Moutan, Cx. Cinnamomi Loureroi (or Cx. Cinnamomi), Rx. Aconiti Lateralis Preparata, Rx. Cyathulae, Sm. Plantaginis |
| Gan-Lu-Yin | 10 | Rx. Rehmanniae, Rx. Rehmanniae Preparata, Hb. Dendrobii, Rx. Asparagi, Rx. Ophiopogonis, Rx. Scutellariae, Hb. Artemisiae Scopariae, Fr. Aurantii, Fol. Eriobotryae, Rx. Glycyrrhizae |
| Zhi-Bai-Di-huang-Wan | 7 | Rx. Anemarrhenae, Cx. Phellodendri, Rx. Rehmanniae Preparata, Fr. Corni Shan, Rx. Dioscoreae, Poria, Cx. Moutan, Rz. Alismatis |
|  |  |  |
| **Single herbs^a^** |  |  |
| Yan-Hu-Suo | 1 | Rhizoma Corydalis |
| Ge-Gen | 1 | Radix Puerariae |
| Dan-Shen | 1 | Radix Salviae Miltiorrhizae |
| Tian-Hua-Fen | 1 | Radix Trichosanthis |
| Jie-Geng | 1 | Radix Platycodi |
| Bei-Mu | 1 | Bulbus Fritillariae Cirrhosae |
| Huang-Qin | 1 | Radix Scutellariae |
| Niu-Xi | 1 | Radix Achyranthis Bidentatae |
| Mai-Men-Dong | 1 | Radix Ophiopogonis |
| Huang-Qi | 1 | Radix Astragali |
| Xuan-Shen | 1 | Radix Scrophulariae |
| Xing-Ren | 1 | Semen Armeniacae |
|  |  |  |
| TCM, traditional Chinese medicine. | | |
| ^a^Information for herbal formulas and single herbs are obtained from the website (http://www.americandragon.com/index.htm). | | |

| **Table B in S1 File. Results of conditional multivariable logistic regression on the occurrence of acute myocardial infarction** | | | | | | |
| --- | --- | --- | --- | --- | --- | --- |
|  | **Acute myocardial infarction** | |  | **Multivariable analysis** | | |
|  | **No (n=1894)** | **Yes (n=66)** |  | **OR** | **(95% CI)** | ***p* value** |
| **CHM user** |  |  |  |  |  |  |
| No | 946 (96.53) | 34 (3.47) |  | 1.00 |  |  |
| Yes | 948 (96.73) | 32 (3.27) |  | 0.68 | (0.35-1.31) | 0.2498 |
| **Liu-Wei-Di-Huang-Wan** |  |  |  |  |  |  |
| No | 1568 (96.43) | 58 (3.57) |  | 1.00 |  |  |
| Yes | 326 (97.60) | 8 (2.40) |  | 0.47 | (0.14-1.57) | 0.2231 |
| **Jia-Wei-Xiao-Yao-San** |  |  |  |  |  |  |
| No | 1583 (96.29) | 61 (3.71) |  | 1.00 |  |  |
| Yes | 311 (98.42) | 5 (1.58) |  | 0.59 | (0.18-1.99) | 0.3982 |
| **Dan-Shen** |  |  |  |  |  |  |
| No | 1569 (96.49) | 57 (3.51) |  | 1.00 |  |  |
| Yes | 325 (97.31) | 9 (2.69) |  | 0.75 | (0.26-2.16) | 0.5942 |
| **Ge-Gen** |  |  |  |  |  |  |
| No | 1561 (96.18) | 62 (3.82) |  | 1.00 |  |  |
| Yes | 333 (98.81) | 4 (1.19) |  | 0.24 | (0.06-0.88) | ***0.0317*** |
| OR, odds ratio; CI, confidence interval; CHM, Chinese herbal medicine. | | | | | | |
| Herbal formula: Liu-Wei-Di-Huang-Wan and Jia-Wei-Xiao-Yao-San; single herb: Dan-Shen and Ge-Gen. | | | | | | |
| Model were adjusted for the covariates included CHM user, age, income, duration from diabetes to hypertension, and comorbidities before hypertension including cardiovascular disease, ischaemic heart disease, chronic kidney disease and hyperlipidaemia (exclusion of acute myocardial infarction). | | | | | | |
| *p* value (*p* < 0.05) was highlighted in bold italic. | | | | | | |
| The ICD-9-CM used for acute myocardial infarction was 410. | | | | | | |

| **Table C in S1 File. Results of conditional multivariable logistic regression on the occurrence of ischemic stroke** | | | | | | |
| --- | --- | --- | --- | --- | --- | --- |
|  | **Ischemic stroke** | |  | **Multivariable analysis** | | |
|  | **No (n=1796)** | **Yes (n=164)** |  | **OR** | **(95%CI)** | ***p* value** |
| **CHM user** |  |  |  |  |  |  |
| No | 899 (91.73) | 81 (8.27) |  | 1.00 |  |  |
| Yes | 897 (91.53) | 83 (8.47) |  | 1.08 | (0.74-1.57) | 0.6868 |
| **Liu-Wei-Di-Huang-Wan** | |  |  |  |  |  |
| No | 1483 (90.87) | 149 (9.13) |  | 1.00 |  |  |
| Yes | 313 (95.43) | 15 (4.57) |  | 0.56 | (0.27-1.16) | 0.1183 |
| **Jia-Wei-Xiao-Yao-San** |  |  |  |  |  |  |
| No | 1501 (90.97) | 149 (9.03) |  | 1.00 |  |  |
| Yes | 295 (95.16) | 15 (4.84) |  | 0.88 | (0.40-1.95) | 0.7528 |
| **Dan-Shen** |  |  |  |  |  |  |
| No | 1487 (91.06) | 146 (8.94) |  | 1.00 |  |  |
| Yes | 309 (94.50) | 18 (5.50) |  | 0.65 | (0.31-1.37) | 0.2576 |
| **Ge-Gen** |  |  |  |  |  |  |
| No | 1481 (90.80) | 150 (9.20) |  | 1.00 |  |  |
| Yes | 315 (95.74) | 14 (4.26) |  | 0.71 | (0.35-1.46) | 0.3538 |
| OR, odds ratio; CI, confidence interval; CHM, Chinese herbal medicine. | | | | | | |
| Herbal formula: Liu-Wei-Di-Huang-Wan and Jia-Wei-Xiao-Yao-San; single herb: Dan-Shen and Ge-Gen. | | | | | | |
| Model were adjusted for the covariates included CHM user, age, income, duration from diabetes to hypertension, and comorbidities before hypertension including cardiovascular disease, ischaemic heart disease, chronic kidney disease and hyperlipidaemia (exclusion of ischemic stroke). | | | | | | |
| *p* value (*p* < 0.05) was highlighted in bold italic. | | | | | | |
| The ICD-9-CM used for ischemic stroke was 433 and 434. | | |  |  |  |  |

| **Table D in S1 File. Results of conditional multivariable logistic regression on the occurrence of hemorrhagic stroke** | | | | | | |
| --- | --- | --- | --- | --- | --- | --- |
|  | **Hemorrhagic stroke** | |  | **Multivariable analysis** | | |
|  | **No (n=1927)** | **Yes (n=33)** |  | **OR** | **(95% CI)** | ***p* value** |
| **CHM user** |  |  |  |  |  |  |
| No | 957 (97.65) | 23 (2.35) |  | 1.00 |  |  |
| Yes | 970 (98.98) | 10 (1.02) |  | 0.43 | (0.14-1.27) | 0.1249 |
| **Liu-Wei-Di-Huang-Wan** | |  |  |  |  |  |
| No | 1594 (98.09) | 31 (1.91) |  | 1.00 |  |  |
| Yes | 333 (99.40) | 2 (0.60) |  | 0.96 | (0.06-14.65) | 0.9765 |
| **Jia-Wei-Xiao-Yao-San** |  |  |  |  |  |  |
| No | 1611 (98.05) | 32 (1.95) |  | 1.00 |  |  |
| Yes | 316 (99.68) | 1 (0.32) |  | 0.73 | (0.04-13.62) | 0.8326 |
| **Dan-Shen** |  |  |  |  |  |  |
| No | 1594 (98.03) | 32 (1.97) |  | 1.00 |  |  |
| Yes | 333 (99.70) | 1 (0.30) |  | 0.81 | (0.06-11.40) | 0.8738 |
| **Ge-Gen** |  |  |  |  |  |  |
| No | 1593 (98.15) | 30 (1.85) |  | 1.00 |  |  |
| Yes | 334 (99.11) | 3 (0.89) |  | 1.22 | (0.15-9.78) | 0.8542 |
| OR, odds ratio; CI, confidence interval; CHM, Chinese herbal medicine. | | | | | | |
| Herbal formula: Liu-Wei-Di-Huang-Wan and Jia-Wei-Xiao-Yao-San; single herb: Dan-Shen and Ge-Gen. | | | | | | |
| Model were adjusted for the covariates included CHM user, age, income, duration from diabetes to hypertension, and comorbidities before hypertension includingcardiovascular disease, ischaemic heart disease, chronic kidney disease and hyperlipidaemia (exclusion of hemorrhagic stroke). | | | | | | |
| *p* value (*p* < 0.05) was highlighted in bold italic. | | | | | | |
| The ICD-9-CM used for hemorrhagic stroke was 430, 431, and 432. | | | | | | |

| **Table E in S1 File. Results of conditional multivariable logistic regression on the occurrence of amputation** | | | | | | |
| --- | --- | --- | --- | --- | --- | --- |
|  | **Amputation** | |  | **Multivariable analysis** | | |
|  | **No (n=1924)** | **Yes (n=36)** |  | **OR** | **(95% CI)** | ***p* value** |
| **CHM user** |  |  |  |  |  |  |
| No | 958 (97.76) | 22 (2.24) |  | 1.00 |  |  |
| Yes | 966 (98.57) | 14 (1.43) |  | 0.44 | (0.15-1.35) | 0.1528 |
| **Liu-Wei-Di-Huang-Wan** | |  |  |  |  |  |
| No | 1592 (98.03) | 32 (1.97) |  | 1.00 |  |  |
| Yes | 332 (98.81) | 4 (1.19) |  | 0.14 | (0.01-1.66) | 0.1189 |
| **Jia-Wei-Xiao-Yao-San** |  |  |  |  |  |  |
| No | 1607 (97.87) | 35 (2.13) |  | 1.00 |  |  |
| Yes | 317 (99.69) | 1 (0.31) |  | ND | ND | ND |
| **Dan-Shen** |  |  |  |  |  |  |
| No | 1591 (97.91) | 34 (2.09) |  | 1.00 |  |  |
| Yes | 333 (99.40) | 2 (0.60) |  | 0.08 | (0.00-2.01) | 0.1238 |
| **Ge-Gen** |  |  |  |  |  |  |
| No | 1592 (97.97) | 33 (2.03) |  | 1.00 |  |  |
| Yes | 332 (99.10) | 3 (0.90) |  | 0.43 | (0.06-3.00) | 0.3912 |
| ND, not determined; OR, odds ratio; CI, confidence interval; CHM, Chinese herbal medicine. | | | | | | |
| Herbal formula: Liu-Wei-Di-Huang-Wan and Jia-Wei-Xiao-Yao-San; single herb: Dan-Shen and Ge-Gen. | | | | | | |
| Model were adjusted for the covariates included CHM user, age, income, duration from diabetes to hypertension, and comorbidities before hypertension including cardiovascular disease, ischaemic heart disease, chronic kidney disease and hyperlipidaemia. | | | | | | |
| *p* value (*p* < 0.05) was highlighted in bold italic. | | | | | | |
| The ICD-9-CM used for amputaion was 84.1 and 84.10-18. | | | | | | |

| **Table F in S1 File. Results of conditional multivariable logistic regression on the occurrence of nephropathy** | | | | | | |
| --- | --- | --- | --- | --- | --- | --- |
|  | **Nephropathy** | |  | **Multivariable analysis** | | |
|  | **No (n=1415)** | **Yes (n=545)** |  | **OR** | **(95% CI)** | ***p* value** |
| **CHM user** |  |  |  |  |  |  |
| No | 719 (73.37) | 261 (26.63) |  | 1.00 |  |  |
| Yes | 696 (71.02) | 284 (28.98) |  | 1.07 | (0.85-1.35) | 0.5498 |
| **Liu-Wei-Di-Huang-Wan** | |  |  |  |  |  |
| No | 1198 (71.39) | 480 (28.61) |  | 1.00 |  |  |
| Yes | 217 (76.95) | 65 (23.05) |  | 0.74 | (0.49-1.13) | 0.1587 |
| **Jia-Wei-Xiao-Yao-San** |  |  |  |  |  |  |
| No | 1175 (70.36) | 495 (29.64) |  | 1.00 |  |  |
| Yes | 240 (82.76) | 50 (17.24) |  | 0.69 | (0.44-1.09) | 0.1131 |
| **Dan-Shen** |  |  |  |  |  |  |
| No | 1175 (70.53) | 491 (29.47) |  | 1.00 |  |  |
| Yes | 240 (81.63) | 54 (18.37) |  | 0.68 | (0.42-1.09) | 0.1084 |
| **Ge-Gen** |  |  |  |  |  |  |
| No | 1169 (70.08) | 499 (29.92) |  | 1.00 |  |  |
| Yes | 246 (84.25) | 46 (15.75) |  | 0.49 | (0.31-0.79) | ***0.0033*** |
| OR, odds ratio; CI, confidence interval; CHM, Chinese herbal medicine. | | | | | | |
| Herbal formula: Liu-Wei-Di-Huang-Wan and Jia-Wei-Xiao-Yao-San; single herb: Dan-Shen and Ge-Gen. | | | | | | |
| Model were adjusted for the covariates included CHM user, age, income, duration from diabetes to hypertension, and comorbidities before hypertension including cardiovascular disease , ischaemic heart disease, chronic kidney disease and hyperlipidaemia (exclusion of nephropathy). | | | | | | |
| *p* value (*p* < 0.05) was highlighted in bold italic. | | | | | | |
| The ICD-9-CM used for nephropathy was 583 and 2504. | | | | | | |

| **Table G in S1 File. Results of conditional multivariable logistic regression on the occurrence of death** | | | | | | |
| --- | --- | --- | --- | --- | --- | --- |
|  | **Death** | |  | **Multivariable analysis** | | |
|  | **No (n=1604)** | **Yes (n=356)** |  | **OR** | **(95% CI)** | ***p* value** |
| **CHM user** |  |  |  |  |  |  |
| No | 757 (77.24) | 223 (22.76) |  | 1.00 |  |  |
| Yes | 847 (86.43) | 133 (13.57) |  | 0.47 | (0.35-0.64) | ***<0.0001*** |
| **Liu-Wei-Di-Huang-Wan** |  |  |  |  |  |  |
| No | 1307 (80.48) | 317 (19.52) |  | 1.00 |  |  |
| Yes | 297 (88.39) | 39 (11.61) |  | 0.43 | (0.25-0.72) | ***0.0015*** |
| **Jia-Wei-Xiao-Yao-San** |  |  |  |  |  |  |
| No | 1310 (79.78) | 332 (20.22) |  | 1.00 |  |  |
| Yes | 294 (92.45) | 24 (7.55) |  | 0.31 | (0.16-0.59) | ***0.0003*** |
| **Dan-Shen** |  |  |  |  |  |  |
| No | 1304 (80.30) | 320 (19.70) |  | 1.00 |  |  |
| Yes | 300 (89.29) | 36 (10.71) |  | 0.39 | (0.22-0.68) | ***0.0008*** |
| **Ge-Gen** |  |  |  |  |  |  |
| No | 1293 (79.67) | 330 (20.33) |  | 1.00 |  |  |
| Yes | 311 (92.28) | 26 (7.72) |  | 0.31 | (0.18-0.55) | ***<0.0001*** |
| OR, odds ratio; CI, confidence interval; CHM, Chinese herbal medicine. | | | | | | |
| Herbal formula: Liu-Wei-Di-Huang-Wan and Jia-Wei-Xiao-Yao-San; single herb: Dan-Shen and Ge-Gen. | | | | | | |
| Model were adjusted for the covariates included CHM user, age, income, duration from diabetes to hypertension, and comorbidities before hypertension including cardiovascular disease, ischaemic heart disease, chronic kidney disease and hyperlipidaemia. | | | | | | |
| *p* value (*p* < 0.05) was highlighted in bold italic. | | | | | | |

| **Table H in S1 File. Regular medical treatment (from diabetes to index day) among type 2 diabetes patients according to CHM usage** | | | | | | |
| --- | --- | --- | --- | --- | --- | --- |
|  | **non-CHM group** | |  | **CHM group** | | ***p* value** |
|  | **(N=979)** | |  | **(N=980)** | |  |
|  | **N** | **%** |  | **N** | **%** |  |
| **Anti-diabetes drug** |  |  |  |  |  |  |
| Biguanides |  |  |  |  |  | ***0.0041*** |
| No | 205 | 20.94 |  | 259 | 26.43 |  |
| Yes | 774 | 79.06 |  | 721 | 73.57 |  |
| Sulfonylureas |  |  |  |  |  | ***0.0009*** |
| No | 143 | 14.61 |  | 199 | 20.31 |  |
| Yes | 836 | 85.39 |  | 781 | 79.69 |  |
| Alpha glucosidase inhibitors |  |  |  |  |  | 0.854 |
| No | 819 | 83.66 |  | 823 | 83.98 |  |
| Yes | 160 | 16.34 |  | 157 | 16.02 |  |
| Thiazolidinediones |  |  |  |  |  | 0.8114 |
| No | 811 | 82.84 |  | 808 | 82.45 |  |
| Yes | 168 | 17.16 |  | 172 | 17.55 |  |
| Insulin |  |  |  |  |  | ***0.0004*** |
| No | 636 | 64.96 |  | 709 | 72.35 |  |
| Yes | 343 | 35.04 |  | 271 | 27.65 |  |
| **Anti-hypertension drug** |  |  |  |  |  |  |
| Antihypertensives |  |  |  |  |  | 1 |
| No | 865 | 88.36 |  | 866 | 88.37 |  |
| Yes | 114 | 11.64 |  | 114 | 11.63 |  |
| Diuretics |  |  |  |  |  | 0.3726 |
| No | 632 | 64.56 |  | 613 | 62.55 |  |
| Yes | 347 | 35.44 |  | 367 | 37.45 |  |
| Beta blocking agents |  |  |  |  |  | ***0.0225*** |
| No | 585 | 59.75 |  | 535 | 54.59 |  |
| Yes | 394 | 40.25 |  | 445 | 45.41 |  |
| Calcium channel blocker |  |  |  |  |  | 0.3147 |
| No | 405 | 41.37 |  | 427 | 43.57 |  |
| Yes | 574 | 58.63 |  | 553 | 56.43 |  |
| ACEI or ARB |  |  |  |  |  | ***0.0027*** |
| No | 356 | 36.36 |  | 422 | 43.06 |  |
| Yes | 623 | 63.64 |  | 558 | 56.94 |  |
| **Anti-hyperlipidemia drug** |  |  |  |  |  |  |
| Statin |  |  |  |  |  | 0.2143 |
| No | 661 | 67.52 |  | 636 | 64.9 |  |
| Yes | 318 | 32.48 |  | 344 | 35.1 |  |
| *p* value (*p* < 0.05) was highlighted in bold italic. | | | | | | |

| **Table I in S1 File. Regular medical treatment (from index day to index day +365) among type 2 diabetes patients according to CHM usage** | | | | | | |
| --- | --- | --- | --- | --- | --- | --- |
|  | **non-CHM group** | |  | **CHM group** | | ***p* value** |
|  | **(N=980)** | |  | **(N=980)** | |  |
|  | **N** | **%** |  | **N** | **%** |  |
| **Anti-diabetes drug** |  |  |  |  |  |  |
| Biguanides |  |  |  |  |  | 0.0548 |
| No | 340 | 34.69 |  | 381 | 38.88 |  |
| Yes | 640 | 65.31 |  | 599 | 61.12 |  |
| Sulfonylureas |  |  |  |  |  | 0.0608 |
| No | 274 | 27.96 |  | 312 | 31.84 |  |
| Yes | 706 | 72.04 |  | 668 | 68.16 |  |
| Alpha glucosidase inhibitors |  |  |  |  |  | 0.3678 |
| No | 855 | 87.24 |  | 868 | 88.57 |  |
| Yes | 125 | 12.76 |  | 112 | 11.43 |  |
| Thiazolidinediones |  |  |  |  |  | 0.5675 |
| No | 839 | 85.61 |  | 830 | 84.69 |  |
| Yes | 141 | 14.39 |  | 150 | 15.31 |  |
| Insulin |  |  |  |  |  | 0.1269 |
| No | 790 | 80.61 |  | 816 | 83.27 |  |
| Yes | 190 | 19.39 |  | 164 | 16.73 |  |
| **Anti-hypertension drug** |  |  |  |  |  |  |
| Antihypertensives |  |  |  |  |  | ***0.0255*** |
| No | 914 | 93.27 |  | 887 | 90.51 |  |
| Yes | 66 | 6.73 |  | 93 | 9.49 |  |
| Diuretics |  |  |  |  |  | 0.6478 |
| No | 709 | 72.35 |  | 718 | 73.27 |  |
| Yes | 271 | 27.65 |  | 262 | 26.73 |  |
| Beta blocking agents |  |  |  |  |  | 0.3032 |
| No | 691 | 70.51 |  | 670 | 68.37 |  |
| Yes | 289 | 29.49 |  | 310 | 31.63 |  |
| Calcium channel blocker |  |  |  |  |  | 0.9279 |
| No | 519 | 52.96 |  | 521 | 53.16 |  |
| Yes | 461 | 47.04 |  | 459 | 46.84 |  |
| ACEI or ARB |  |  |  |  |  | ***0.0011*** |
| No | 432 | 44.08 |  | 504 | 51.43 |  |
| Yes | 548 | 55.92 |  | 476 | 48.57 |  |
| **Anti-hyperlipidemia drug** |  |  |  |  |  |  |
| Statin |  |  |  |  |  | 0.2384 |
| No | 733 | 74.8 |  | 710 | 72.45 |  |
| Yes | 247 | 25.2 |  | 270 | 27.55 |  |
| *p* value (*p* < 0.05) was highlighted in bold italic. | | | | | | |

**The supporting information:**

The cell line work (BCRC number:60127) used to create the charts in Fig. 3A and 3B was purchased from Food Industry Research and Development Institute in Taiwan (https://catalog.bcrc.firdi.org.tw/BSAS_cart/controller?event=SEARCH&bcrc_no=60127&type_id=4&keyword=smooth;;muscle;;cells). These cells were derived from the thoracic aorta of rats and served as a commonly used model of vascular smooth muscle cells [1] and were approved by the Animal Care and Use Committee (IACUC) of China Medical University, Taichung, Taiwan.

1. Bhadriraju K, Elliott JT, Nguyen M, Plant AL. Quantifying myosin light chain phosphorylation in single adherent cells with automated fluorescence microscopy. BMC Cell Biol. 2007;8:43. Epub 2007/10/19. doi: 1471-2121-8-43 [pii]

10.1186/1471-2121-8-43. PubMed PMID: 17941977; PubMed Central PMCID: PMC2213650.
